# The Relationship Between Neutrophil-Lymphocyte and Platelet-Lymphocyte Ratios With Hospital Stays and Mortality in the Emergency Department

**DOI:** 10.7759/cureus.12179

**Published:** 2020-12-20

**Authors:** Mustafa Cifci, Huseyin C Halhalli

**Affiliations:** 1 Emergency Medicine, University of Health Sciences, Kocaeli Derince Training and Research Hospital, Kocaeli, TUR

**Keywords:** acute pancreatitis, neutrophil/lymphocyte ratios, platelet/lymphocyte ratios

## Abstract

Background

Most acute pancreatitis scoring is made in the first 48-72 hours or later. Like many inflammatory processes, Neutrophil/lymphocyte ratio (NLR) and platelet/lymphocyte ratio (PLR) can be useful in showing the severity and extent of inflammation in acute pancreatitis. Our study aimed to evaluate whether these rates affect mortality according to the NLR and PLR values ​​of patients diagnosed with acute pancreatitis by examining the blood samples taken within the first hour after admission to the emergency department rates are useful in predicting the length of stay.

Methods

In our retrospective study, 557 patients applied to our clinic for 4.5 years, whose amylase and lipase values ​​were higher than two times the cut-off value in blood tests and whose CT imaging was compatible with acute pancreatitis were included in the study.

Results

The median length of hospitalization of the patients was 4.0 (3.0-6.0) days. Gallstones were detected in 320 (57.5%) patients. Mortality of less than a year was observed in 45 (8.1%) of the study population. Eighteen of the patients (3.2%) showed the need for follow-up in the intensive care unit. A statistically significant relationship was found between mortality and variables hematocrit (HCT), red cell distribution width (RDW),c-reactive protein CRP), glucose, urea, potassium, albumin, PLR, and NLR (p <0.05). A statistically significant correlation was observed between RDW, NLR, glucose, and CRP levels in the two groups divided according to the median value of 4 days we found on hospitalization (p <0.05). According to the graphics and test results obtained by ROC analysis, the mortality status can be predicted at a statistically significant level with PLR and NLR diagnostic tests (p <0.05).

Conclusion

High levels of NLR, PLR, RDW, glucose, CRP, urea, potassium, low albumin and hematocrit values ​​at the first admission in the Emergency Service seem to be associated with increased 1-year mortality in acute pancreatitis.

## Introduction

Acute pancreatitis is an inflammatory process with mortality varying between 3% and 17% [1,2]. Because of these high mortality rates, different scoring systems have been developed based on clinical and laboratory findings, radiological risk factors, severity grading systems, and various serum markers [3]. While some of these scorings are done when the patient is admitted immediately to help the patient's triage, some are evaluated in the first 48-72 hours or later [4]. While the sensitivity is between 40-90% in the Ranson Scoring System, 56% sensitivity was observed in BISAP (Bedside Index for Severity in Acute Pancreatitis) [5,6]. The Modified Glasgow 2 scoring has a 56-85% sensitivity in evaluating the severity of both alcohol-related and biliary pancreatitis [7]. Even if all these scoring systems' sensitivity and specificity in predicting the severity of acute pancreatitis vary between 55% and 90% according to the cut-off values of the parameters used and the scoring timing, these scoring must complete at least 48 hours to apply. Also, the complexity of the scoring limits their use [8].

Neutrophil/lymphocyte ratio (NLR) and platelet/lymphocyte ratio (PLR) have been accepted as useful indicators to predict prognosis and survival in various cancers that show inflammatory processes [9]. Like many inflammatory processes, NLR and PLR can be useful in showing the severity and extent of inflammation in acute pancreatitis. Since the severity of the disease in acute pancreatitis is related to the inflamed pancreatic tissue size, the NLR and PLR ratios may also be useful in demonstrating this relationship. In our study, we aimed to evaluate whether these rates affect mortality according to NLR and PLR values of patients diagnosed with acute pancreatitis by examining the blood samples taken within the first hour after admission to the emergency department and whether these rates are useful in predicting the length of stay.

## Materials and methods

Study population

Our study was carried out in a 3rd level academic emergency medicine clinic to which approximately 400000 patients apply annually. Our study was conducted among patients diagnosed with acute pancreatitis after admission to the emergency department.

Study design

Our study is a retrospective study. By examining our hospital's computer-based data system, patients who applied to our clinic for 4.5 years between 01.01.2015-01.07.2019, amylase and lipase values higher than two times the cut-off value in blood tests and CT imaging compatible with acute pancreatitis were detected. Demographic data (age, gender), blood amylase, lipase, glucose, albumin, lactate dehydrogenase (LDH), aspartate aminotransferase (AST), alanine aminotransferase (ALT), c-reactive protein (CRP), urea, creatinine (CRE), sodium (Na), potassium (K), chlorine (Cl), calcium (Ca) levels, haemoglobin in the blood (HGB), hematocrit (HCT), erythrocyte count (RBC), mean platelet volume (MPV), erythrocyte distribution range (RDW), leukocyte (LEU), neutrophil (NEU), platelet (PLT), lymphocyte (LNF) counts, Neutrophil / Lymphocyte count ratio (NLR), Platelet / Lymphocyte count ratio (PLR) were determined. Besides, the duration of hospital stay, intensive care needs, bile ducts and gallstones, and one-year mortality after diagnosis were determined. Patients who did not have sufficient information in the data system (with or without blood tests or imaging findings) left the hospital voluntarily, patients with haematological malignancies referred to other hospitals, and patients under the age of 18 were excluded from the study. Only the first applications of the patients with more than one application were evaluated.

Statistical analysis

The data's compliance with normal distribution was evaluated by a histogram, q-q graphs, and Shapiro-Wilk test. Variance homogeneity was tested with the Levene test. In comparison between paired groups, an independent two-sample t-test was used for quantitative variables. The normality assumption was provided, and Mann Whitney U test was used for those who did not provide normality assumption. Chi-square analysis was used to compare categorical variables. ROC charts were obtained by applying ROC (Receiver Operating Characteristic) analysis to evaluate the diagnostic test performance in determining the mortality status of Platelet / Lymphocyte and Neutrophil / Lymphocyte diagnostic tests. Sensitivity, selectivity, positive predictive value, and negative predictive values were calculated for the cut-off values determined according to the Youden method. The data analysis was carried out in TURCOSA (Turcosa Analytics Ltd Co, Turkey, www.turcosa.com.tr) statistics software. The significance level was accepted as p <0.05.

Ethics committee approval

The data collected were collected for study purposes only. Local Ethics Committee approval was obtained for the research on 12.03.2020 with the protocol number 2020-29.

## Results

Five hundred eighty-nine patients were diagnosed with acute pancreatitis after admission to the emergency service between 01.01.2015 and 01.07.2019. Five hundred fifty-seven of these were included in the study. Sixteen patients left the hospital voluntarily, 12 patients were transferred to another hospital, and four patients were excluded due to hematological malignancy diagnosis. The average age of 557 observations obtained in the study was 58.66 ± 17.30; 315 of them (56.6%) were women. The patients' median length of hospitalization was found to be 4.0 (3.0-6.0) days. Gallstones were detected in 320 (57.5%) patients. Mortality of less than one year was observed in 45 (8.1%) of the study population. Eighteen (3.2%) of the patients showed the need for follow-up in the intensive care unit. The median of Ranson score of observations in the study was 1.0 (1.0-2.0), and the Ranson score of 131 (23.5%) was three and above (Table 1). Laboratory variables related to observations are shown in Table 1. The median, first, and third quartile values for PLR and NLR were 156.7 (103.2-223) and 4.6 (2.8-8.2), respectively (Table 1).

**Table 1:** Baseline characteristics of the patients with acute pancreatitis ICU, intensive care unit; ALT, alanine aminotransferase; AST, aspartate aminotransferase; LDH, lactate dehydrogenase; Na, Sodium; K, Potassium; Ca, calcium; CRP, C reactive protein; WBC, white blood cell; RBC, red blood cell; Hct; Hematocrit; RDW. red cell distribution width; SD, standard deviation.

	Mean	SD
Age (Years)	58.66	17.30
Garden (Male, %)	242 (%43.4)	
Total Hospital Stay (Day)	4.0	4.16
ICU Stay (Day) Yes No	18 (%3.2) 539 (%96.8)	
Gallstone (%) Yes No	320 (%57.5) 237 (%42.5)	
Mortality (%) 1 year <1 year	512 (%91.9) 45 (%8.1)	
RANSON Skore <3 3	426 (%76.5) 131 (%23.5)	
Laboratory Data		
Glucose (mg/dL)	127.0	66.16
Urea (mg/dL)	29.0	25.39
Creatinine (mg/dL)	0.9	0.86
ALT (U/L)	99.0	182.61
AST (U/L)	121.0	210.50
LDH (U/L)	313.0	305.56
Albumin (g/dL)	4.02	0.39
Amylase (U/L)	912.0	1365.49
Lipase (U/L)	1672.0	3313.35
Na (mEq/L)	138.71	3.27
K (mEq/L)	4.10	0.42
CPR (mg/dL)	6.8	40.61
WBC (L)	10687.97	4179.04
RBC (10^6^L)	4.33	0.56
HCT (%)	38.96	4.64
RDW (%)	15.75	2.22
Platelets (L)	246544.34	82606.56
Neutrophil (L)	7400.0	3975.25
Lymphocyte(L)	1500.0	1326.34
Platelets/lymphocyte ratio	156.7	149.25
Neutrophile/lymphocyte ratio	4.6	7.72

Age, urea, AST, ALT, LDH, NEU, LNF, NLR, and PLR values ​​were compared in the data divided into two groups according to the Ranson score received at the first admission to the emergency service. As a result of these comparisons, age, urea, AST, ALT, LDH, NEU, LNF, PLR, and NRL variables were found to be statistically significantly different in the groups with Ranson score below three and above 3 (p <0.05) (Table 2).

**Table 2:** Comparison of patients with Ranson score <3 vs. patients with Ranson score ≥3 AST, aspartate aminotransferase; ALT, alanine aminotransferase; LDH, lactate dehydrogenase.

	RANSON		p
	RANSON <3	RANSON ≥3	
	(n=426)	(n=131)	
Age (Years)	55.65±17.47	68.45±12.47	<0.001
Urea (mg/dL)	29.0(25.0-33.0)	31.0(27.0-45.0)	0.002
AST (U/L)	79.0(27.0-173.0)	362.0(264.0-518.0)	<0.001
ALT (U/L)	60.0(22.0-168.0)	250.0(159.0-406.0)	<0.001
LDH (U/L)	276.5(219.0-355.0)	530.0(424.0-655.0)	<0.001
Neutrophil (​L)	7000.0(5000.0-9400.0)	8600.0(6000.0-12700.0)	<0.001
Lymphocyte (L)	1600.0(1200.0-2300.0)	1300.0(800.0-2000.0)	<0.001
Platelets/lymphocyte ratio	151.8(98.3-213.0)	190.5(119.4-275.7)	<0.001
Neutrophile/lymphocyte ratio	4.3(2.5-7.1)	6.8(3.7-11.7)	0.001

Mortality was similar according to gender distribution, and there was no statistically significant difference (p> 0.05). No statistically significant relationship was found between the groups formed according to the mortality time and the variables of emergency admission Ranson score, WBC, RBC, MPV, PLT, NEU, LNF, Na, AST, ALT, LDH, CRE, Amylase, Lipase (p> 0.05). There was no statistically significant difference between the presence of obstruction and mortality (p> 0.05).

A statistically significant correlation was found between mortality and variables HCT, RDW, CRP, glucose, urea, potassium, albumin, PLR, and NLR (p <0.05) (Table 3). A statistically significant relationship was observed between the need for intensive care hospitalization and PLR, NLR, Glucose, CRP, and Albumin levels (p <0.05) (Table 4). A statistically significant correlation was observed between RDW, NLR, glucose, and CRP levels in the two groups that we divided according to the median value of 4 days, which we found on hospitalization (p <0.05) (Table 4).

**Table 3:** Comparison of patients with mortality ICU, intensive care unit; CRP, C reactive protein; Hct; Hematocrit; RDW. red cell distribution width; SD, standard deviation.

	Mortality		
	>1 years (n=512)	≤ 1 years (n=45)	
Age (Years)	57.39±16.9	73.04±15.33	<0.001
Garden (Male, %)	224(43.8)	18(40.0)	0.627
Hospital Stay (Day)	4.0(3.0-6.0)	6.0(4.0-11.0)	<0.001
ICU Stay			<0.001
Yes	4(0.8)	14(31.1)	
No	508(99.2)	31(68.9)	
Gallstone			0.560
No	216(42.2)	21(46.7)	
Yes	296(57.8)	24(53.3)	
RANSON Score			0.071
<3	397(77.5)	29(64.4)	
≥3	115(22.5)	16(35.6)	
Laboratory Data			
Glucose (mg/dL)	126.5(108-153.5)	151(117-221)	<0.001
Urea (mg/dL)	29.0(25.0-33.0)	41.0(26.0- 60.0)	<0.001
Albumin (g/dL)	4.05±0.35	3.67±0.60	<0.001
CPR (mg/dL)	6.3(2.0- 25.6)	12.4(6.3- 50.7)	0.002
HCT (%)	39.1±4.54	37.33±5.49	0.041
RDW (%)	15.66±2.10	16.79±3.11	0.001
Platelets (L)	246984.38± 83920.62	241537.78± 66405.41	0.672
Neutrophil (L)	7400.0(5200.0-9900.0)	7900.0(6200.0-11200.0)	0.090
Lymphocyte(L)	1600.0(1100.0-2300.0)	1200.0(800.0-1600.0)	0.063
Platelets/lymphocyte ratio	154.7(101.2-214.9)	227.0(129.0-295.7)	0.004
Neutrophile/lymphocyte ratio	4.5(2.7-7.9)	6.6(3.8-11.5)	0.004

**Table 4:** Comparison of patients with hospitality CRP, C reactive protein; RDW, red cell distribution width; SD, standard deviation.

	Hospital Stay (Day)					ICU Stay (Day)				
	≤4 day (n=300)		>4 day (n=227)		p	Yes (n=18)		No (n=539)		p
	Mean	SD	Mean	SD		Mean	SD	Mean	SD	
Age (Years)	57.24	17.74	60.32	16.65	0.036	63.44	21.56	58.5	17.14	0.290
Glucose (mg/dL)	136.69	60.73	154.36	70.93	0.00005	214.61	112.04	142.51	62.92	0.010
Albumin (g/dL)	4.04	0.31	4.00	0.46	0.92104	3.53	0.76	4.03	0.36	0.010
Amylase (U/L)	1338.48	1206.66	1505.27	1527.72	0.36047	1032.17	1487.66	1428.24	1360.85	0.07
Lipase (U/L)	3367.70	3151.73	3549.77	3496.23	0.84796	1939.17	2067.24	3502.22	3336.38	0.421
CPR (mg/dL)	17.94	28.41	31.23	50.40	0.00017	60.67	87.58	22.86	37.62	0.026
RDW (%)	15.59	2.35	15.94	2.05	0.01881	15.83	1.77	15.75	2.23	0.726
Neutrophil (L)	7659.33	3568.08	8594.16	4356.50	0.02094	10005.56	6065.28	8026.72	3878.41	0.214
Lymphocyte (L)	1840.00	1105.05	1750.97	1546.02	0.09462	1561.11	1382.3	1806.86	1325.03	0.052
Platelets/lymphocyte ratio	176.63	120.42	207.94	175.83	0.05951	226.06	111.23	189.91	150.3	0.042
Neutrophile/lymphocyte ratio	6.14	6.30	7.79	9.03	0.00427	9.9	7.38	6.8	7.72	0.024

**Figure 1:**
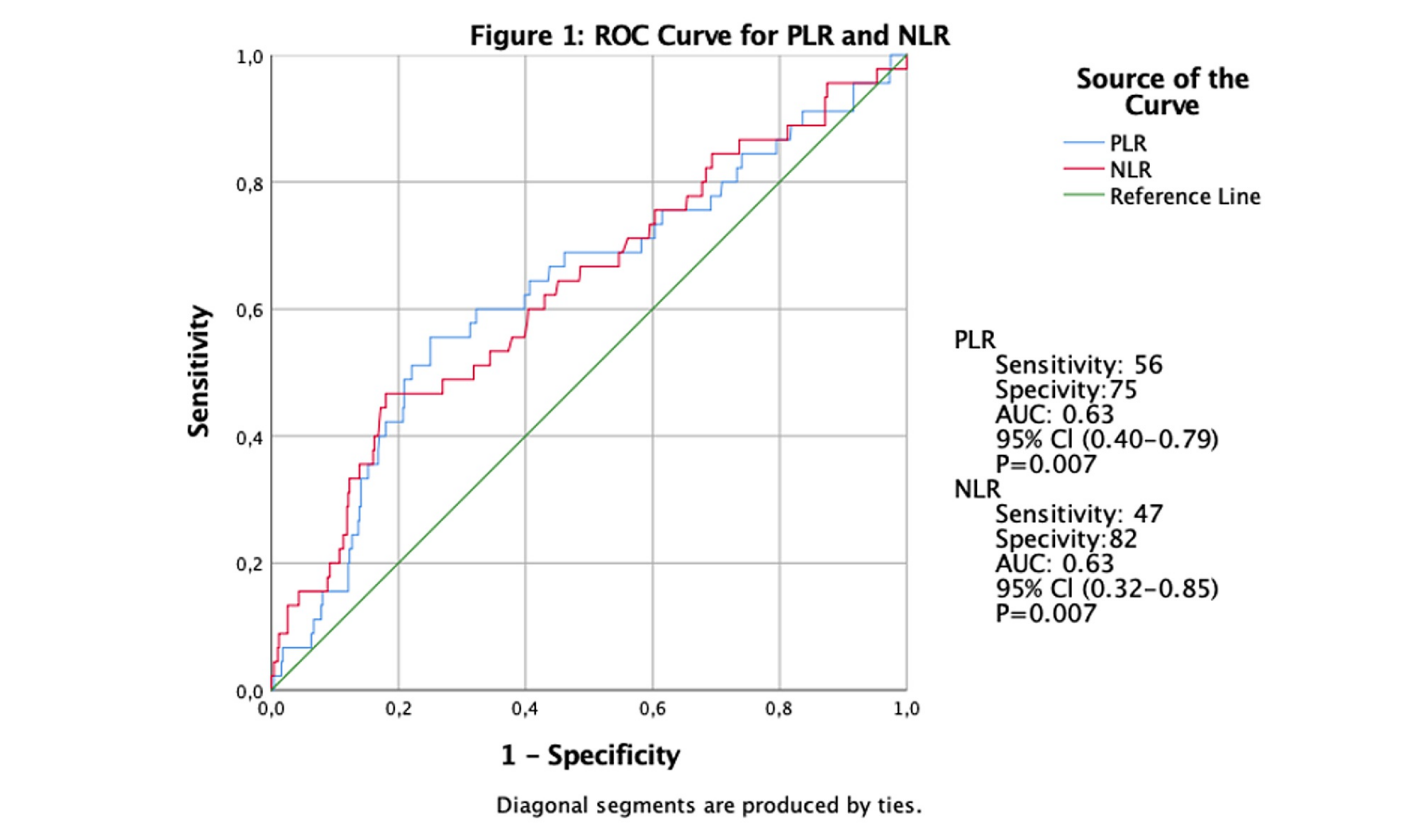
ROC curve for PLR and NLR for one-year mortality in patients with acute pancreatitis PLR: Platelet / Lymphocyte count ratio, NLR:  Neutrophil / Lymphocyte count ratio, AUC: Area under the curve, ROC: Receiver operating characteristic, CI: Confidence interval)

According to the graphics and test results obtained by ROC analysis, mortality can be predicted at a statistically significant level with PLR and NLR diagnostic tests (p <0.05) (Figure 1).

Performance criteria obtained according to the cut-off value determined for Platelet / Lymphocyte (215) are given in Table 5. The probability of the observation with a mortality period of 1 year or less from two randomly selected observations according to the platelet/lymphocyte ratio is one year or less according to the observation with a mortality duration of 1 year or more. The sensitivity of Platelet / Lymphocyte test; In the case of illness, the test's probability of giving a positive result is 56%. Platelet / Lymphocyte test; In the absence of disease, there is a 75% chance that the test will give a negative result. Positive predictive value of Platelet / Lymphocyte test; If the test is positive, the probability of developing the disease is 16%. Negative predictive value of the Platelet / Lymphocyte test; In cases where the test is negative, the probability of not developing the disease is 95%. Performance criteria obtained according to the cut-off value (≥9.09) determined for NLR are given in Table 5. The AUC value, according to the NLR at the cut-off value determined, is 62.8%. The specificity of NLR was determined as 47%, and its sensitivity was 82%.

**Table 5:** Diagnostic test performance criteria obtained to determine mortality status with Platelet / Lymphocyte and Neutrophil / Lymphocyte diagnostic tests SEN, sensitivity; SPE, specificity; PPV, positive predictive value; NPV, negative predictive value; AUC, area under curve, ROC, receiver operating characteristic, CI, confidence interval

Variables	Statistical diagnostic measures				ROC curve statistic	
	SEN (95%CI)	SPE (95%CI)	PPV (95%CI)	NPV (95%CI)	AUC	p
Platelet / Lymphocyte (³215)	0.56 (0.40-0.70)	0.75 (0.71-0.79)	0.16 (0.14-0.27)	0.95 (0.91-0.96)	0.628	0.007
Neutrophil / Lymphocyte (³9.09)	0.47 (0.32-0.62)	0.82 (0.78-0.85)	0.18 (0.15-0.30)	0.94 (0.90-0.96)	0.628	0.007

## Discussion

Acute pancreatitis is an inflammatory disease with severe morbidity and mortality. The incidence of acute pancreatitis, which is currently diagnosed frequently in Emergency Departments, has increased over the last 25 years [10,11]. Also, diagnosis and treatment cause high costs [12]. For this reason, it may be useful to predict the severity of the disease and make risk stratification in Emergency Units with a large patient population. However, most scoring systems can be calculated 24 to 48 hours after hospitalization. Scoring systems, where the first blood tests taken in the emergency department can calculate the severity of the disease and the complications that may develop in a short time, can contribute to the provision of better care for patients with acute pancreatitis.

A statistically significant relationship was observed between NLR, PLR, RDW, and 28-day mortality in the study of Zhou et al., which included 472 patients in 2019 to predict acute pancreatitis disease severity and prognosis in the early period [13]. Although our study was conducted with a different population than this study and evaluated the 1-year mortality, it has similarities with this study statistically. A statistically significant correlation was found in our study in terms of 1-year mortality in patients with increased NLR, PLR, and RDW levels (p <0.005).

The effect of impaired glucose tolerance on acute pancreatitis has not been fully elucidated [14]. Kikuta et al., in a study conducted in 2015 to show the role of impaired glucose tolerance in the prognosis of acute pancreatitis, it was observed that the mortality rates of Diabetes Mellitus (DM) patients diagnosed with acute pancreatitis were higher than those without. It was predicted that impaired glucose tolerance might be useful in the severity of acute pancreatitis [15]. There is no study in the literature regarding the effect of glucose levels in the emergency department on 1-year mortality in patients diagnosed with acute pancreatitis. In our study, a statistically significant relationship was found between high glucose levels and 1-year mortality in patients diagnosed with acute pancreatitis (p <0.001). In this respect, our study is original in terms of blood glucose levels and long-term mortality assessment in patients with acute pancreatitis.

In acute pancreatitis, hemoconcentration occurs with a fluid loss to the third space, and accordingly, HCT elevation is observed, and this is an indicator of poor prognosis for acute pancreatitis [16]. Remes-Troche et al., in a study conducted in 2005, it was shown that HCT is a poor predictor of acute pancreatitis prognosis [17]. From the recent studies on the effect of HCT levels on mortality in patients diagnosed with acute pancreatitis, Zhou et al. and Jino et al. in 2019 draw attention [13,18]. In these studies, a statistical significance was found between high HCT and BUN levels and mortality. In our study, a significant relationship was found between high urea levels and mortality, supporting previous studies (p <0.001). However, in our study, contrary to these studies, low HCT levels were found to be associated with increased mortality (p = 0.041). This may be due to the heterogeneity of the patients included in the studies.

CRP is one of the acute phase reactants synthesized in the liver in response to interleukin-1 and interleukin-6 after an inflammatory stimulus. As a result of meta-analysis, 24-48 of CRP in acute pancreatitis. Although it is known that the levels measured per hour have a higher rate of accuracy than the levels seen during the first application to the emergency department in terms of mortality, it is thought that the blood values checked at the first application are also significant in terms of mortality [19]. In our study, by the literature, we observed a significant relationship in terms of one-year mortality with CRP levels obtained during the first admission to the emergency department (p = 0.002).

Potassium levels play an essential role in cell membrane electrophysiology, and abnormalities in their levels can lead to malignant arrhythmias [20]. According to the results obtained after meta-analyses, high potassium levels in patients are the only risk factors for mortality, and end-stage renal failure in the subsequent periods [21]. There is no study in the literature evaluating potassium levels and subsequent mortality in patients with acute pancreatitis. In our study, a significant relationship was found with high potassium levels in terms of one-year mortality. The reason for this may be fluid loss in the third space and tissue necrosis observed in acute pancreatitis.

Albumin is an essential modulator of plasma oncotic pressure and an essential transporter of endogenous and exogenous ligands. It is also an important indicator of nutritional status [22]. In a recent study conducted by Hong et al. In 2017, which included 700 patients, low albumin levels in patients with acute pancreatitis were associated with persistent organ failure alone [23]. In our study, a statistically significant result was found between low albumin levels in blood values obtained at first admission and one-year mortality in patients diagnosed with acute pancreatitis in the emergency department (p <0.001). Our study supports the literature in this respect.

Several studies in the literature draw attention to the relationship between the blood tests obtained at the first admission in patients diagnosed with acute pancreatitis in the emergency department with hospitalization duration and the need for intensive care. In the study by O'Connell et al., 185 patients were included, a statistical significance was observed between RDW and NLR and the duration of hospital stay [24]. In this study, the average length of stay in hospital was 7.65 days, the need for ICU was 8.1%, and mortality was 1.08%. In our study, the average duration of hospital stay was four days, the need for ICU was 3.2%, and the mortality was 8.2%. However, this study was based on 40-day values in terms of mortality. However, similar to the study by O'Connell et al., In our study, a statistically significant relationship was found between RDW and NLR and the duration of hospital stay. In this respect, our work is compatible with the work of O'Connell et al.

In the study conducted by Azab et al. in 2011, NLR's effect on intensive care admission and hospital stay duration was examined [25]. In this study, the patients were divided into three groups. The relationship between intensive care admissions and the number of days of hospitalization with NLR was evaluated, and statistically significant results were found in both cases. Although the patients were not divided into groups in our study, similar results were obtained with the study by Azab et al. A significant relationship was found between increased NLR in ICU admissions and hospital stay length.

Our study found a statistically significant relationship between high glucose and CRP levels in blood tests taken at the first admission and hospitalization duration and the ICU's need. Also, we observed that low albumin levels and increased PLR correlated with the need for ICU admission. There is no study evaluating the relationship of these values with ICU admissions and length of hospital stay. However, as mentioned above, each of PLR, glucose, and CRP is essential in acute pancreatitis [13-15,19,22,23]. Therefore, we think that PLR, glucose, and CRP are associated with hospital stay length and ICU admissions.

Many studies have tried to calculate cut-off values by dividing patient populations into groups. In our study, the ROC curve was used to calculate the cut-off value. Supiah et al., similarly, in their study in 2013, the ROC curve was used instead of groups to calculate the cut-off value [26]. In this study, the relationship between NLR and severe acute pancreatitis was evaluated, and the cut-off value for NLR at first admission was found to be 10.6. Zhou et al., in their study, patients were divided into three groups and evaluated by looking at 28-day mortality. In this study, the total mortality rate was found to be 3.45%, the cut-off value for NLR was 12.19, and for PLR, it was 148.85 [13]. In our study, 1-year mortality was evaluated, and when the cut-off value for NLR was 9.09 and 215 for PLR, the mortality rate was found to be 8.1%. There is no other study examining first-admission NLR and PLR cut-off values and 1-year mortality in acute pancreatitis. More studies are needed to determine a more reliable value in cut-off values.

Limitations 

Our study is a single-center retrospective study. Repeating it with more extensive multi-center studies may be beneficial to obtain more reliable data. Blood tests in different ethnic groups may yield different results since they are performed on patients of a limited ethnic group

## Conclusions

High levels of NLR, PLR, RDW, glucose, CRP, urea, potassium, low albumin and hematocrit values at the first admission in the Emergency Department seem to be associated with increased 1-year mortality in acute pancreatitis. High levels of NLR, RDW, glucose, and CRP at the first admission in the emergency department appear to be associated with an increased hospital stay, increased NLR, PLR, glucose, and CRP values low albumin values in acute pancreatitis.
